# Intraparenchymal Chordoma in the Brain Stem: A Review of Surgical Management and Case Highlight

**DOI:** 10.7759/cureus.67937

**Published:** 2024-08-27

**Authors:** Malek Bashti, Long Di, Manav Daftari, Emade Jaman, Tyler Cardinal, Michael W Robinson, James V Boddu, Adib Abla

**Affiliations:** 1 Neurological Surgery, University of Miami Miller School of Medicine, Miami, USA

**Keywords:** surgical management, skull base, brain stem, chordoma, intraparenchymal tumor

## Abstract

We present a rare case of an intraparenchymal chordoma in the brain stem of a 69-year-old male with a history of multiple chordoma recurrences. Chordomas are uncommon tumors that originate from notochordal remnants, with intraparenchymal presentations in the brain stem being particularly rare. A 69-year-old male with a history of clival chordoma three years after primary endoscopic resection and adjuvant proton-beam radiotherapy and a recurrence one year postoperatively for which he underwent a second surgery, presented with severe headaches, weakness, diaphoresis, and difficulty ambulating. Head CT in the ER revealed a 2.7 x 3.5 cm hyperdense lesion in the pons, indicating acute hemorrhage. Magnetic resonance imaging (MRI) suggested a hemorrhagic radiation-induced cavernoma. A right retrosigmoid craniotomy was performed, and the lesion was resected without major complications. Final pathology reported an intraparenchymal hemorrhagic chordoma. To our knowledge, this is the first case of intra-axial chordoma, particularly in the brain stem. It highlights the importance of considering intraparenchymal chordoma on the differential when evaluating for recurrence versus other treatment-induced pathologies and changes. This may prompt the neurosurgeon to reconsider treatment options and weigh the risks of watchful waiting versus biopsy or even aggressive surgical management.

## Introduction

Chordoma, a rare and formidable pathology, is a slow-growing bone sarcoma of the neuroaxis, manifesting in one of 1,000,000 people annually [1]. Originating from remnants of the notochord, chordomas exhibit a predilection for the axial skeleton. Despite commonly presenting with well-circumscribed borders that can be amenable to gross total resection, a high recurrence rate increases overall mortality. Five-year overall survival with a new diagnosis of chordoma is 50% but can be raised to as high as 65% in patients with tumor-free resection margins [2]. Proton beam radiotherapy has recently become a pivotal treatment for chordoma by enabling higher radiation doses directly to tumors with minimized risk to surrounding tissues [3].

The treatment of chordoma necessitates a multidisciplinary approach; surgical resection with negative margins remains paramount for accessible sites. Adjuvant radiation therapy, including proton beam and stereotactic radiosurgery, is crucial for managing residual disease or as a primary modality when surgery is not feasible. For systemic control, chemotherapy has traditionally had a limited role due to poor response; however, the advent of targeted therapies and immunotherapies has opened new avenues for treatment. Agents targeting specific molecular pathways identified in chordoma pathogenesis, such as platelet-derived growth factor receptor (PDGFR), epidermal growth factor receptor (EGFR), and mammalian target of rapamycin (mTOR) pathways, are under investigation and hold promise for improving outcomes in patients with metastatic disease​​ [4].

Here, we present an exceedingly rare case report of an intraparenchymal chordoma in a 69-year-old male with a history of recurrent clival chordoma. The patient presented with a pontine mass initially suspected to be a radiation-induced cavernoma. Surprisingly, surgical intervention revealed an intraparenchymal chordoma, a finding that not only challenges existing paradigms but also enriches our understanding of chordoma pathophysiology and clinical behavior, paving the way for revisiting and refining diagnostic and therapeutic paradigms.

## Case presentation

Preoperative course

**Figure 1:**
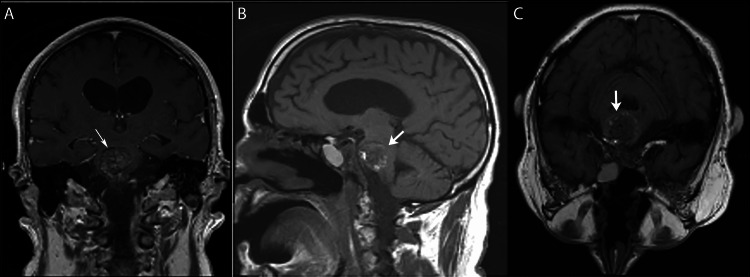
Pre-operative MRI T1 scans in coronal (A), sagittal (B), and axial (C) views demonstrate the recurrent lesion. The MRI series shows a T1/T2 heterogeneous enhancing lesion in the pons with associated vasogenic edema, initially thought to be secondary to hemorrhagic cavernoma. A heterogeneously enhancing clival lesion extends into the retroclival and prevertebral regions, encasing the basilar artery, suggesting residual/recurrent chordoma.

A 69-year-old male, with a history of clival chordoma status post-endoscopic endonasal resection in 2000, multiple rounds of prior proton beam radiotherapy, most recently in September 2017, and retrosigmoid craniotomy for the resection of the recurrence in 2021, presented with a two-day history of severe headaches, diffuse weakness, and difficulty ambulating. A neurologic exam was significant for baseline left hemibody weakness - which was a residual deficit from his 2021 surgery - but was otherwise non-focal. CT of the brain demonstrated a 2.7 x 3.5 cm well-circumscribed hyperdensity in the pons that was increased in size compared to a prior study in 2021, concerning acute hemorrhage. A follow-up MRI with and without contrast demonstrated a T1/T2 heterogenous enhancing lesion within the pons, with extensive susceptibility signal and surrounding vasogenic edema. The preliminary diagnosis was a hemorrhagic radiation-induced cavernoma (Figure 1).

Surgical technique

**Figure 2:**
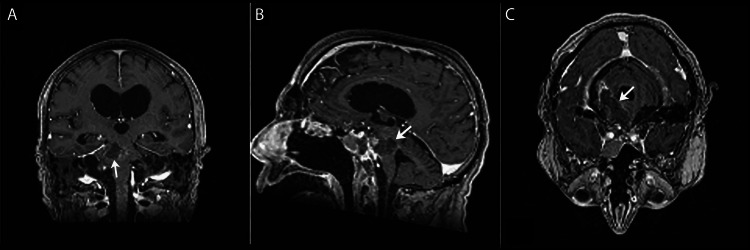
Postoperative MRI T1 scans in coronal (A), sagittal (B), and axial (C) views. These post-operative T1-weighted MRI scans confirm the absence of residual chordoma.

The patient was taken to the operating room for a redo right retrosigmoid craniotomy. An infra-trochlear, trans-quadrangular lobule approach was used to access the lesion that had arisen on the pial surface at the pontine midbrain junction. The malformation was visualized as a large, hemorrhagic malformation measuring approximately 3 cm. The lesion was removed with a standard microsurgical technique. The anterior portion of the lesion was fibrous, likely adhering to the basilar artery, and resected without breaking through the anterior pial surface. The lesion was dissected at the superior, inferior, anterior, and posterior borders and sent to pathology. The surgery was otherwise uncomplicated, and the dura and skin were closed in the usual fashion (Figure 2).

Pathological examination

**Figure 3:**
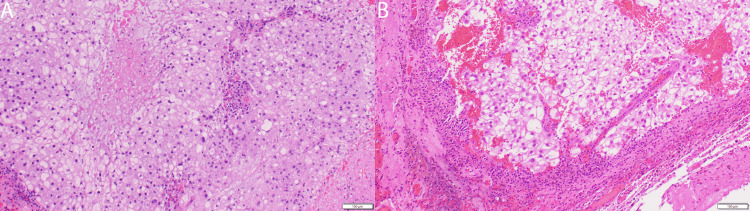
Histopathological examination of chordoma. Microscopic images of the specimen from the patient’s prior surgery for recurrence in 2021 showcase the diagnostic histopathological features of chordoma large polyhedral cells with eosinophilic cytoplasm and characteristic physaliphorous cells, noted for their cytoplasmic vacuolation. Chronic inflammatory infiltrate and focal necrosis areas further confirm a conventional type chordoma diagnosis, emphasizing cellular heterogeneity and post-treatment pathological landscape (3A). Examination of the lesion resected in November 2023 reveals similar histopathologic characteristics with the added significant finding of extensive intratumoral hemosiderin deposits. These deposits, indicative of previous intratumoral hemorrhage, mark an advanced stage of pathological progression characterized by the tumor's hemorrhagic activity (3B).

Pathological examination confirmed a conventional type chordoma, displaying large polyhedral cells with eosinophilic cytoplasm, physaliphorous cells with variable cytoplasmic vacuolation, chronic inflammatory infiltrate, and focal necrosis. In addition, compared with samples from prior surgeries, there were now pronounced intratumoral hemosiderin deposits, suggesting a history of intratumoral hemorrhage (Figures 3A-3B).

Postoperative course

Postoperatively, the patient was uneventfully extubated but exhibited left-sided hemiplegia with purposeful movements only in the right hemibody and was not initially following commands. On postoperative day (POD) three, the patient exhibited a worsening level of consciousness requiring re-intubation. Repeat CT was unremarkable with only expected post-operative findings and mild hydrocephalus; MRI showed no acute ischemia or worsening edema. The patient gradually improved to spontaneous eye opening and following commands and was re-extubated on POD14. Unfortunately, the patient exhibited progressively worsening hypoxic respiratory failure despite non-invasive therapies complicated additionally by active aspiration pneumonia; on POD39, the patient’s family elected to discontinue any further aggressive therapies and pursued comfort measures only, and the patient expired on POD40.

## Discussion

Literature review

Our case report of an intraparenchymal chordoma in a 69-year-old male represents a rare and complex presentation in neurosurgery. The unique histological features of this chordoma deviate from the common extradural type, highlighting the diagnostic challenges and the necessity of tailored surgical approaches in such atypical cases. The employment of advanced imaging techniques was pivotal in revealing the elusive nature of this intraparenchymal chordoma and tailoring a patient-specific surgical intervention. A literature review was performed to further understand the classification, pathophysiology of metastasis, and surgical management of chordomas. A summary of the findings can be found in Table 1.

**Table 1:** Summary of the key findings from the literature review. Summarized from the following references: 1,4-6,8,11-15,20

Topic	Findings
Classification of Chordomas	Rare, low-grade malignancy from notochordal remnants. Global incidence: 0.08 to 0.5 per 100,000 persons. Three subtypes: conventional, poorly differentiated, and undifferentiated. Molecular characteristics: T gene (brachyury) duplication, CDKN2A/B loss, EGFR amplification.
Challenges in Treatment	Late presentation, local recurrence, poor response to conventional therapies. Surgical resection and advanced radiation therapy (proton beam) are primary treatments. Limited role of chemotherapy; targeted therapies and immunotherapies are promising.
Molecular Pathogenesis	Common genetic alterations: T gene (brachyury) duplication, CDKN2A/B loss. EGFR amplification. Pathways: EGFR, PDGFβ, IGFR1, mTOR, MET, PI3K. Poorly differentiated chordomas: marked by SMARCB1 deletion and loss of INI1 expression.
Metastasis	Rare, predominantly to lungs. Median survival for metastatic disease: 130.4 months. Mechanisms: aberrant cell cycle regulation, receptor tyrosine kinase expression. Rapid development to distant bones linked to aggressive behavior.
Surgical Management	Lawton's taxonomy: six subtypes of pontine BSCMs with distinct anatomical locations and surgical approaches. Tailored surgical interventions based on lesion subtype.
Our Case Example	First case of intra-axial chordoma in the brain stem. Highlights diagnostic challenges and the importance of advanced imaging techniques. Emphasizes the necessity of tailored surgical approaches for atypical cases.

Classification of chordomas

Chordoma, a rare and low-grade malignancy originating from notochordal remnants, presents unique challenges in diagnosis and management due to its indolent growth, propensity for local recurrence, and poor response to conventional therapies. The global incidence of chordomas ranges from 0.08 to 0.5 per 100,000 persons and primarily affects the skull base, mobile spine, and sacrum [1]. Patients usually present with midline pain, sometimes with radicular features due to nerve root compression. Therein lies a critical need for advanced diagnostic and treatment strategies to improve patient outcomes.

However, these tumors are challenging to treat because they often present late in the disease course, encapsulate adjacent neurovascular anatomy, seed resection cavities, recur locally, and respond poorly to radiotherapy and conventional chemotherapy. Surgical resection, coupled with advancements in radiation therapy such as focused photon and proton beam radiation, remains the cornerstone of treatment, although the role of chemotherapy is evolving with ongoing research into the molecular underpinnings of these tumors [5,6].

The histological and molecular diversity of chordomas, with three primary subtypes identified, further complicates their management. The majority are conventional chordomas, with a minority presenting as either poorly differentiated or dedifferentiated, each associated with distinct prognostic implications. Conventional chordomas, constituting approximately 95% of cases, include a subtype known as chondroid chordoma. The very rare and poorly differentiated chordomas are characterized by cohesive sheets of epithelioid cells, eosinophilic cytoplasm, loss of INI1 staining, and positive brachyury expression. The least common, dedifferentiated chordomas, represent less than 1% of cases and are noted for their biphasic nature, combining conventional chordoma with high-grade sarcomatous transformation, typically manifesting as high-grade undifferentiated pleomorphic sarcoma or osteosarcoma, and are associated with poor prognosis​​ [4].

Molecular characterization of chordomas reveals a complex genetic landscape. Notably, T gene (brachyury) duplication is observed in approximately 27% of sporadic cases, and nearly all notochordal tumors overexpress brachyury due to epigenetic mechanisms. Other frequent genetic alterations include homozygous or heterozygous loss of CDKN2A or CDKN2B at 9p21 and EGFR amplification. Chordomas commonly activate pathways such as EGFR, PDGFβ, IGFR1, IGF1, mTOR, MET, and PI3K and involve chromatin remodeling genes such as ARID1A, PBRM1, and SETD2. A subset of sporadic chordomas exhibits chromothripsis, a genetic mechanism resulting in extensive gene rearrangements. Interestingly, poorly differentiated chordomas are molecularly distinct, marked by SMARCB1 deletion and subsequent loss of INI1 expression​​ [7].

Metastasis of intraparenchymal chordomas

Our investigation into the literature revealed a notable gap in studies on intraparenchymal chordomas, contrasting with the documented cases of metastatic chordomas. Metastasis, though rare, predominantly affects the lungs and is more prevalent among younger patients, particularly those with local recurrences. The median survival time from initial diagnosis for patients with metastatic disease is approximately 130.4 months, significantly less than that for patients without metastasis (159.3 months). The prognosis of metastatic chordoma is highly dependent on the primary tumor location and the site of metastasis, with metastasis to distal bone developing most rapidly and associated with the worst prognosis ​​​​[8,9].

The pathophysiology of chordoma metastasis is not well-documented; however, given their origin from notochordal remnants and their unique histological features, including physaliphorous cells in a myxoid or chondroid matrix, the mechanism might involve the aberrant expression of proteins such as cytokeratin, EMA, S100, and brachyury, which are common in chordomas​​ [10]. Many molecular mechanisms have been investigated in the metastatic progression of chordomas, including chromosomal instability, DNA methylation, and miRNA expression; however, two important underlying mechanisms are aberrant cell cycle regulation and receptor tyrosine kinase expression [11]. Yakkioui et al. found overexpression of mouse double murine 2 (MDM2) in 56% of chordoma tumors that results in elevated levels of truncated p53 protein and loss of function. As a result, the mutant form of p53 is unable to inhibit cell-cycle regulator cyclin-dependent kinase 4 (CDK-4) [12]. Receptor tyrosine kinases (RTKs) have been found in downstream signaling pathways leading to overexpressed secondary messengers, play an important role in the tumorigenesis of chordomas, and serve as potential pharmacologic targets [13]. Additionally, the propensity for metastasis and its rapid development, particularly to distant bones, suggests an aggressive behavior in certain forms of chordoma, possibly influenced by histological differentiation and local recurrence rates [4].

The complex pathophysiology of chordoma metastasis, involving aberrant cell cycle regulation and receptor tyrosine kinase expression, underscores the importance of comprehensive molecular profiling in developing effective therapeutic strategies. Molecular therapy targets being investigated include several receptor tyrosine kinases (e.g., PDGFR, EGFR), downstream cascades (e.g., PI3K/Akt/mTOR), brachyury (a transcription factor unique to chordoma), and the FGF/MEK/ERK pathway. Molecular insights, particularly the duplication of the T gene (brachyury) and alterations in CDKN2A/B, EGFR, and chromatin remodeling genes, have paved the way for targeted therapies, highlighting the potential of personalized medicine in improving chordoma treatment paradigms and reserving aggressive surgery for only the most advanced cases [14,15].

Chordomas predominantly metastasize to the lungs, liver, bones, and lymph nodes, with a less frequent yet significant occurrence of intraparenchymal brain metastases, which are particularly rare and challenging to manage​​ [16]. The propensity for chordomas to metastasize intraparenchymally might be influenced by several factors, including the genetic landscape of the tumor. For instance, mutations in the TBXT gene, a known susceptibility gene for chordoma, might play a role in the tumor's aggressive behavior and its ability to metastasize to rare locations such as the brain parenchyma [17].​ Additionally, the genetic dependencies identified in chordoma, such as those revealed through CRISPR-Cas9 screening by Sharifnia et al. highlighted potential vulnerabilities that could influence metastatic behavior such as the known chordoma dependency, TBXT (T; brachyury), and a range of additional dependencies, including PTPN11, ADAR, PRKRA, LUC7L2, SRRM2, SLC2A1, SLC7A5, FANCM, and THAP1 genes [18].

Surgical management of intraparenchymal chordomas

In addressing the surgical approaches to pontine lesions, a significant advancement can be seen in the taxonomy developed by Catapano et al., which categorizes brain stem cavernous malformations (BSCMs) based on their clinical and radiological features [19]. This taxonomy, derived from a comprehensive study of 601 patients undergoing microsurgical resection of BSCMs over three decades, provides a detailed framework for understanding these complex and variable lesions. Specifically, pontine BSCMs were subdivided into six subtypes: basilar, peritrigeminal, middle peduncular (MP), inferior peduncular, rhomboid, and supraolivary. The first three subtypes exhibit distinct anatomical locations and associated neurological deficits, which include contralateral hemiparesis in basilar lesions, sensory changes in peritrigeminal lesions, and contralateral hemisensory loss with ipsilateral ataxia in MP lesions [19].

The taxonomy not only enhances the understanding of these lesions but also informs surgical strategy. For instance, basilar lesions are optimally approached via the pterional craniotomy and anterior transpetrosal approach, while peritrigeminal lesions are best accessed through the extended retrosigmoid craniotomy and transcerebellopontine angle approach. Similarly, MP lesions are approached via the extended retrosigmoid craniotomy and trans-middle cerebellar peduncle approach. This stratification of pontine BSCMs according to their anatomical and clinical characteristics allows for tailored surgical interventions, potentially leading to improved outcomes, as evidenced by the favorable results observed in a majority of the patients in this study [19].

Furthermore, the comprehensive nature of this taxonomy, as detailed in both parts of Lawton's publication underscores the importance of individualized treatment planning in neurosurgical practice, particularly for complex lesions such as pontine BSCMs [19,20]. This approach could be especially relevant in rare or atypical cases, such as intraparenchymal chordomas, where the standard treatment paradigms may not be directly applicable.

## Conclusions

In conclusion, chordomas represent a complex clinical entity requiring a multidisciplinary approach to management. Our study not only adds to the understanding of chordoma biology and treatment strategies but also underscores the importance of integrating advanced imaging and molecular insights into patient-specific treatment planning, contributing valuable knowledge to the sparse literature on intraparenchymal chordomas and emphasizing the ongoing need for innovation in neurosurgical practice.
